# RETRACTED: Ahmed et al. Thermal Analysis of a Metal–Organic Framework ZnxCo_1-X_-ZIF-8 for Recent Applications. *Polymers* 2021, *13*, 4051

**DOI:** 10.3390/polym16243596

**Published:** 2024-12-23

**Authors:** Moustafa Ahmed, Yas M. Al-Hadeethi, Ahmed Alshahrie, Arwa T Kutbee, Essam R. Shaaban, Ahmed F. Al-Hossainy

**Affiliations:** 1 Physics Department, Faculty of Science, King Abdulaziz University, P.O. Box 80203, Jeddah 21589, Saudi Arabiayoutobhyt@yahoo.com (Y.M.A.-H.); asertun33@gmail.com (A.A.); w.dridi.ph@gmail.com (A.T.K.); 2 Physics Department, Faculty of Science, Al-Azhar University, Assiut 11751, Egypt; esam_ramadan2008@yahoo.com; 3 Chemistry Department, Faculty of Science, New Valley University, Kharga 11765, Egypt

The journal retracts the article “Thermal Analysis of a Metal–Organic Framework ZnxCo1-X-ZIF-8 for Recent Applications” [1], which is cited above.

Following publication, concerns were brought to the attention of the publisher regarding a partial overlap of images presented in this article [1].

Adhering to our complaint procedure, an investigation was conducted that confirmed a partial duplication with Figure 6. While the authors cooperated with the Editorial Office during the investigation, they were unable to satisfactorily explain the partial overlap nor account for anomalies present in the Supplementary Materials provided.

As a result, the Editorial Board has lost confidence in the reliability of the findings and has decided to retract this publication [1], as per MDPI’s retraction policy (https://www.mdpi.com/ethics#_bookmark30), accessed on 22 August 2024.

This retraction was approved by the Editor-in-Chief of the journal *Polymers*.

The authors state that they disagree with the decision to retract the paper and that they confirm the validity of all the results and figures contained in the paper according to the experimental results and analyses they have obtained.

## References

[1] Ahmed M., Al-Hadeethi Y.M., Alshahrie A., Kutbee A.T., Shaaban E.R., Al-Hossainy A.F. (2021). RETRACTED: Thermal Analysis of a Metal–Organic Framework ZnxCo_1-X_-ZIF-8 for Recent Applications. Polymers.

